# Q Fever Endocarditis: A Challenging Diagnosis in a Patient Referring Weight Loss and Anorexia, and With Granulomas in Gastric Biopsy Specimens

**DOI:** 10.1002/ccr3.9635

**Published:** 2024-11-29

**Authors:** Ana Elena Lago‐Rodriguez, Pablo Arturo Fraile‐Ribot, Isabel Amengual‐Antich, Francisco Gual‐Capllonch, Mercedes Garcia‐Gasalla

**Affiliations:** ^1^ Cardiology Department Hospital Universitario Son Espases Palma Illes Balears Spain; ^2^ Microbiology Department Health Research Institute of the Balearic Islands (IdISBa), Hospital Universitario Son Espases Palma Illes Balears Spain; ^3^ Pathology Department, Hospital Universitario Son Espases Palma Illes Balears Spain; ^4^ Internal Medicine Department, Facultad de Medicina, Health Research Institute of the Balearic Islands (IdISBa) Hospital Universitario Son Espases, Universitat de Ses Illes Balears Palma Illes Balears Spain

**Keywords:** *Coxiella burnetii*, endocarditis, gastric granulomas, Q fever, weight loss

## Abstract

Diagnosis of Q fever endocarditis is challenging since clinical findings are non‐specific and diagnosis is mainly made by indirect methods such as serology. A progressive constitutional syndrome, severe asthenia, anorexia with no fever and histopathological findings of non‐necrotizing gastric granulomas in a gastric biopsy were found preceding a cardiac failure in our case report. Prolonged treatment with doxycycline and hydroxychloroquine is mandatory, and cardiac valve surgery may be needed.

## 1 Introduction

*Coxiella burnetii* is an intracellular gram‐negative bacterium that causes Q fever, a worldwide zoonotic disease affecting people mainly in rural areas. The primary route of transmission to humans is through inhalation of aerosols containing the pathogen, especially those formed from placental derivatives. The reservoirs are wild or domestic mammals and ticks. Human infection can present as acute Q fever or chronic Q fever [1]. Symptomatic acute Q fever occurs in approximately half of the infected subjects. The clinical presentation is diverse, from a mild influenza‐like symptomatic condition to pneumonia or a prolonged fever with abnormal liver function tests. Chronic Q fever accounts for 1%–5% of all reported Q fever cases. The chronic presentations are endocarditis, infected aneurysms or vascular prostheses, or osteomyelitis. Endocarditis presents non‐specifically with negative blood cultures. It occurs almost exclusively in patients who have a preexisting valve disease or who are immunocompromised.

Diagnosis is mainly made by indirect methods such as serology—antibodies are expressed against phase II antigens during the acute infection and against phase I antigens during the chronic infection—or by direct methods such as *C. burnetii* –specific PCR of serum or tissue samples.

## 2 Case History/Examination

A 59‐year‐old patient was referred in April 2023 to our internal medicine outpatient clinic in Son Espases hospital in Mallorca (Spain). He manifested severe asthenia and reported a 30‐kg weight loss together with the presence of a metallic taste leading to a loss of appetite that began in December 2022 after a flu‐like syndrome. At that time, the patient had been previously diagnosed with hypertension and colonic diverticulosis and was receiving treatment with olmesartan. He was a trade worker and he referred more than 30 trips to China in the past with prolonged stays in that country.

Physical examination revealed a body temperature of 36°C, a blood pressure of 110/63 mmHg, and a pulse of 85 beats per minute, and the remainder of the examination was normal.

## 3 Methods (Differential Diagnosis, Investigations, and Treatment)

Several diagnostic tests were conducted. Hematologgical and biochemical analyzes were performed, and elevated liver enzymes were noticed (Table 1) Thyroid function tests revealed a TSH of 0.17 IU/mL with normal T3 and T4. A proteinogram showed hypergammaglobulinemia, with an IgG of 1580 mg/dL (500–1200), a monoclonal IgG kappa and B2‐microglobulin of 10,100 μg/L (800–2500). Complement C3, C4, and CH50 were in the normal range, and the rheumatoid factor and antinuclear antibodies were both negative.

**TABLE 1:** Laboratory examinations on admission.

White cell	4640/μL
Hemoglobin	13.4 g/dL
Platelet	140,000/μL
VSG	7 mm
PCR	1.16 mg/dL
Bilirubin	< 1.2 mg/dL
GOT	404 U/L
GPT	298 U/L
GGT	118 U/L
ALP	131 U/L
Glucose	96 mg/dL
Creatinine	1.51 mg/dL

**FIGURE 1:**
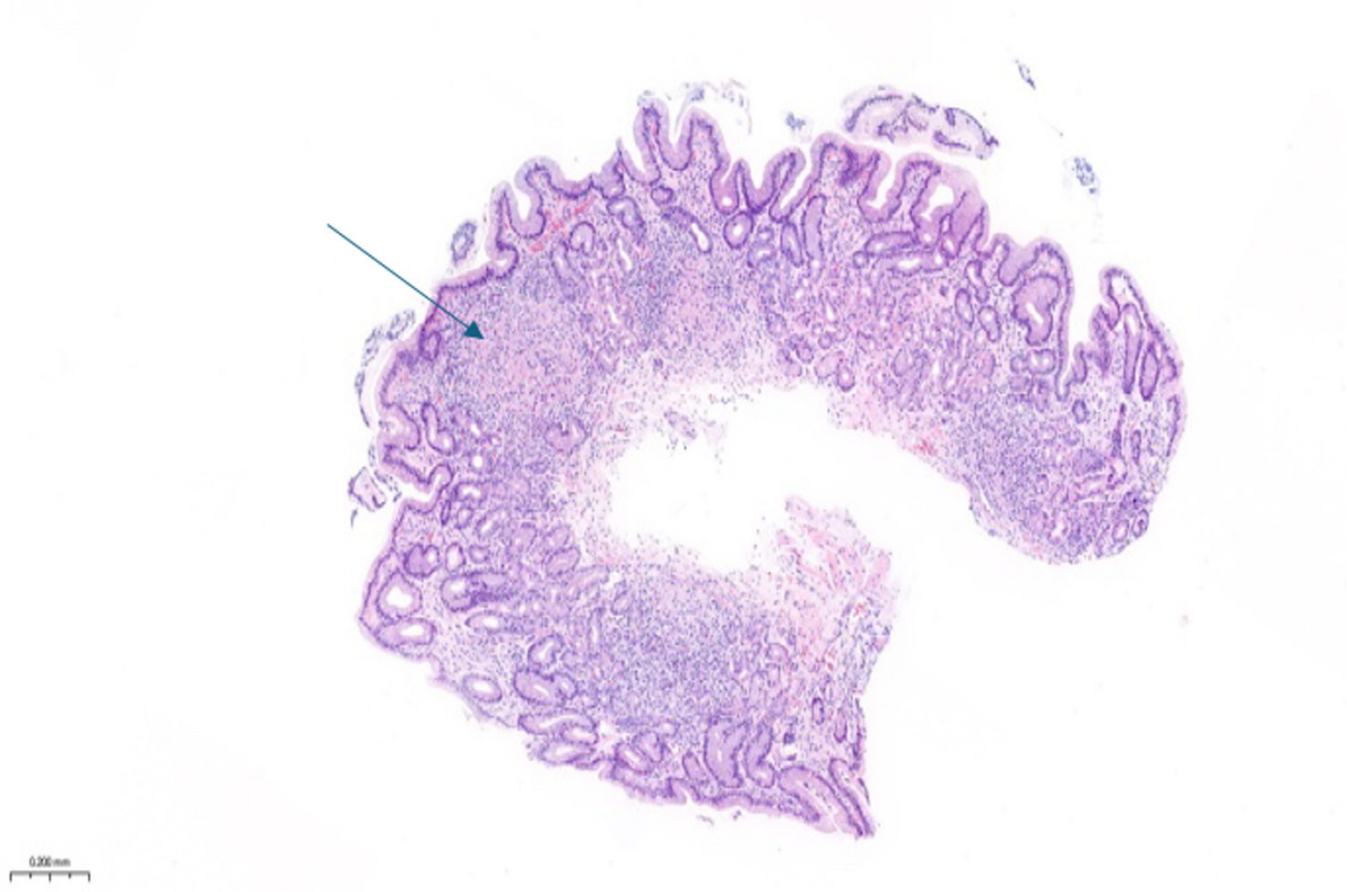
Histopathological image from a gastric biopsy specimen showing non‐necrotizing granulomas, round collection of lymphocytes, histiocytes and multinucleated giant cells (arrow).

Serological tests for HIV, hepatitis B and C, and syphilis were negative, and IgG *C. burnetii* was found to be positive with a titre of 1/512 in May and of 1/2048 in July 2023, together with a negative IgM result. Both a full body CT scan and a PET scan were found to be normal except for a colonic diverticulosis and a small inguinal hernia. Bone marrow aspiration did not provide evidence of lymphoproliferative syndrome or neoplasia. A gastroscopy and a colonoscopy were performed, revealing an erosive antral gastritis. The histopathological study was diagnostic for a non‐necrotizing granulomatous gastritis, with negative staining for *Helicobacter pylori* (Figure 1).

In August 2023, the patient's clinical condition had deteriorated as he reported a weight loss of 40 kg, asthenia, and anorexia, but no chest pain, dyspnoea, fever or any other symptoms. He was diagnosed with acute Q fever and monoclonal gammopathy of undetermined significance (MGUS), and an extrapulmonary sarcoidosis diagnosis was considered.

A 2‐week doxycycline treatment was prescribed for acute Q fever, treatment with mycophenolate was started for a suspected atypical sarcoidosis, and a cardiac ultrasound was requested.

**FIGURE 2:**
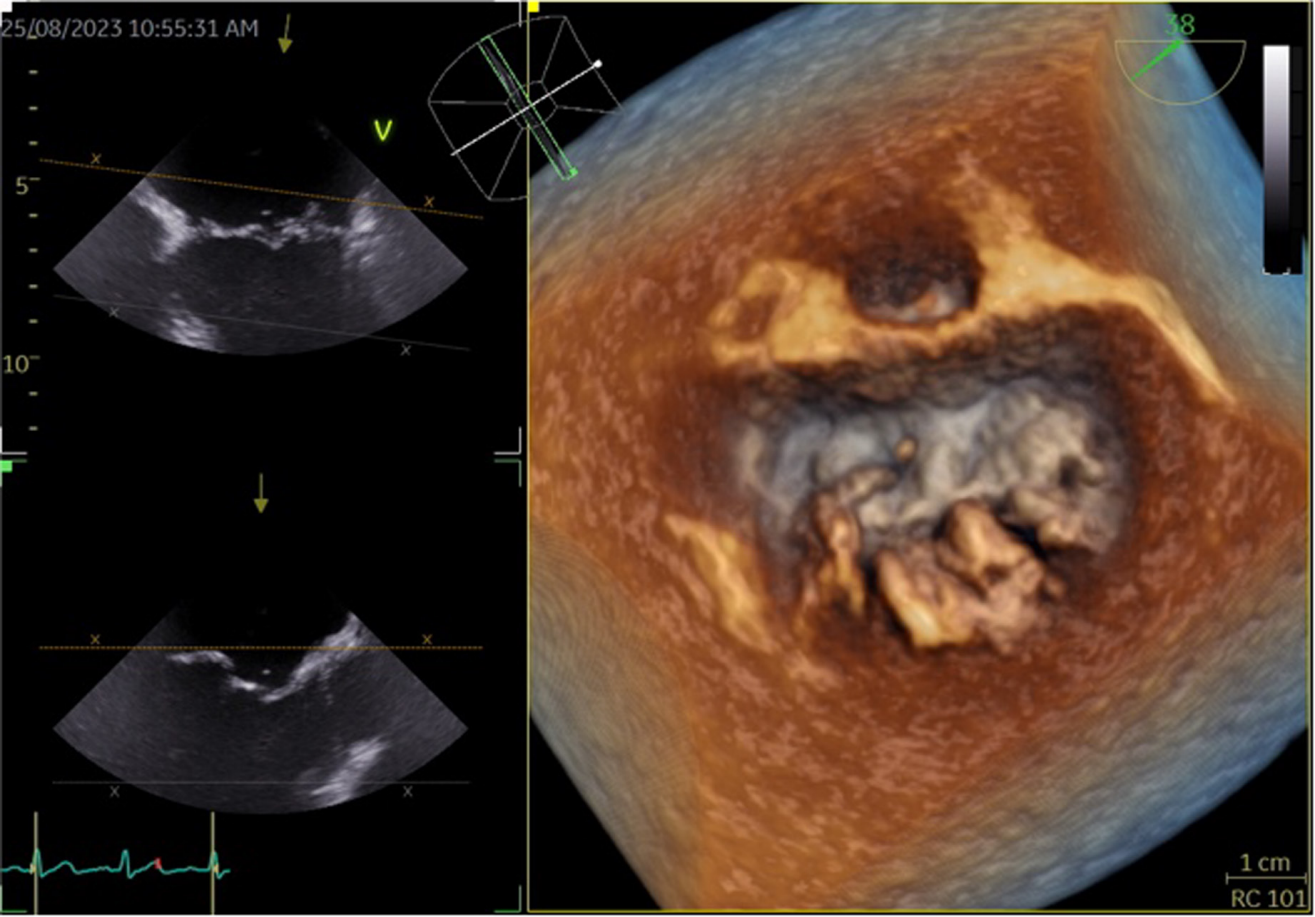
Mitral valve reconstruction from a three‐dimensional (3D) transesophageal echocardiography suggestive of vegetation in the posterior leaflet.

**FIGURE 3:**
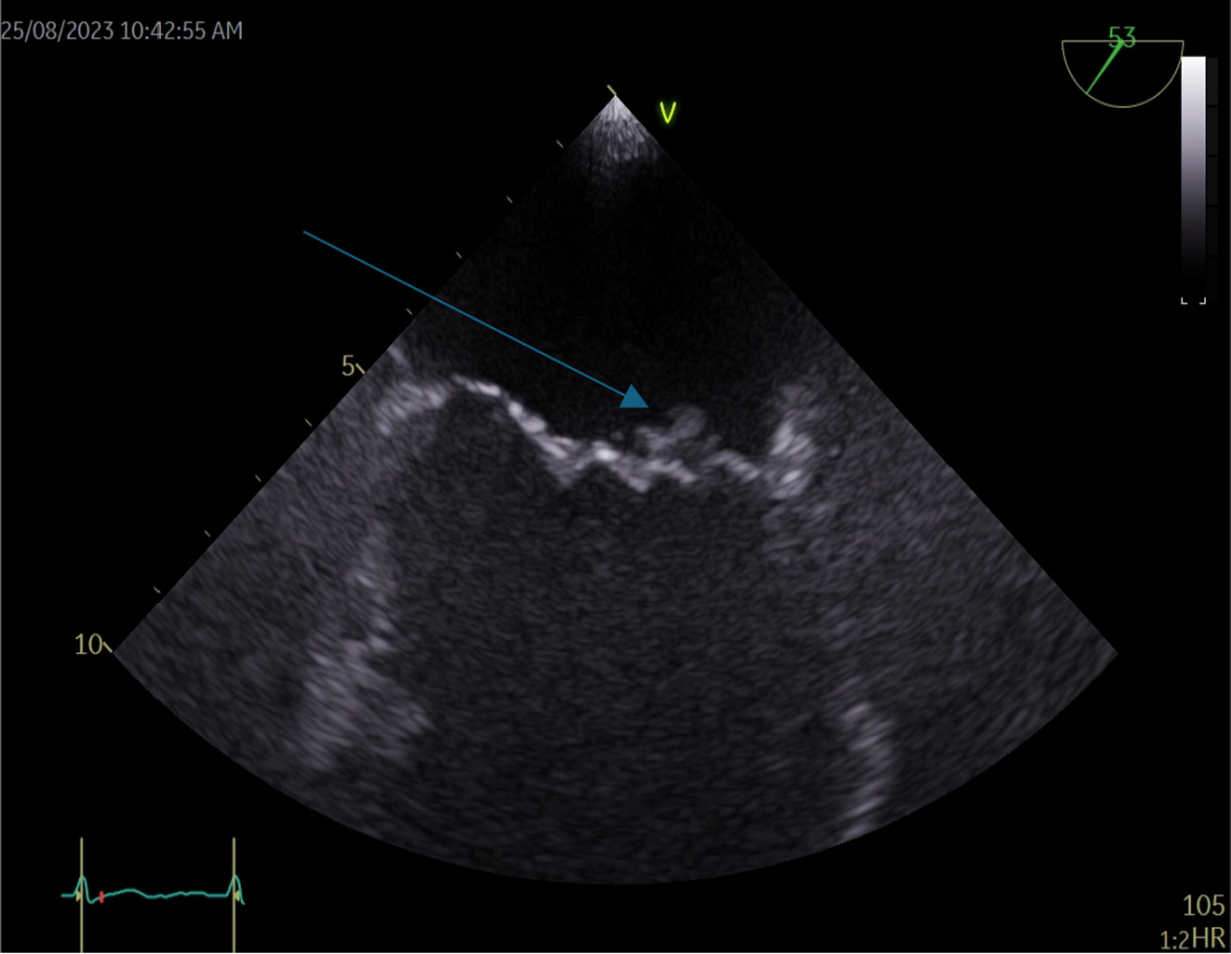
Longitudinal plane of the mitral valve from a transesophageal two‐chamber view delineating the left atrium in the upper part, the left ventricle in the lower part, and the mitral valve separating the two chambers: transesophageal echocardiography image suggestive of a vegetation in the posterior velum.

Two weeks after treatment initiation at his last visit, the patient went to the emergency room of our hospital reporting a worsening of asthenia, dyspnoea, and orthopnoea in the last 4 days. He was admitted to the internal medicine department for a suspected heart failure. A transthoracic echocardiography (TTE) was performed, revealing severe mitral insufficiency with prolapse of both mitral leaflets and a thickening of the right leaflet with a doubtful image of vegetation. A transesophageal echocardiography (TEE) demonstrated the presence of vegetation on the posterior leaflet of the mitral valve (Figures 2 and 3). Blood culture was negative for bacterial growth.

## 4 Conclusion and Results (Outcome and Follow‐Up)

At this point, the clinical case and the different diagnostic tests performed were reviewed and a blood culture‐negative endocarditis was suspected. Phase 1 and phase 2 *Coxiella* serological tests were conducted, and IgG titres of 1/16.384 (Phase 1) and 1/4.096 (Phase 2) were found. The patient fulfilled two major diagnostic criteria from the 2023 European Society of Cardiology and a definite Q fever endocarditis was diagnosed [2]. The granulomas observed in the histopathological study of the gastric biopsy were considered to be possibly related to the Q fever.

The patient started on Q fever endocarditis treatment: doxycycline 100 mg twice daily and hydroxychloroquine 200 mg three times a day and was discharged.

In the outpatient clinic follow‐up visit, asthenia and anorexia had improved and the patient had started to gain weight but he referred progressive worsening dyspnea. An echocardiogram performed in January 2024 showed a left ventricular systolic disfunction with ejection fraction by Simpson of 55% (being the previous in August 2023 70%) and a mitral valve with a myxoid appearance with thickening and prolapse of both leaflets and pansystolic mitral insufficiency. Therefore, the patient was presented to the medical‐surgical committee and he finally underwent mitral valve surgery in July 2024. A mitral valve repair was performed, using a P2 string and annuloplasty with implantation of a Physio II #38 ring. Unfortunately, no tissue specimen was taken for a microbiological molecular diagnosis.

In August 2024, the IgG phase 1 titre for *C. burnetii* had decreased to 1/2.048.

## 5 Discussion

We describe the case of a patient with a chronic Q fever infection with an onset characterized by a constitutional syndrome in which a rapidly progressive asthenia stood out. Diagnosis of chronic Q fever is challenging and is often delayed primarily due to non‐specific symptoms and unfamiliarity with chronic Q fever. However, early diagnosis has important implications, as chronic Q fever causes high morbidity and mortality [3, 4]. Although endocarditis is the main manifestation of chronic Q fever, it should be noted that only 1%–5% of cases evolve from acute to chronic Q fever. Preexisting heart valve disease, aortic aneurysm, vascular grafts, immunocompromised status, and pregnancy are reported risk factors for the development of chronic Q fever.

In the case presented, the patient came to the clinic with a constitutional syndrome without any other symptoms. Weight loss has rarely been described as a symptom of Q fever. Moreover, this patient did not have any known predisposing valvopathy and systolic murmurs were not detected on repeated physical examinations during the first months of the disease, which is why an echography was not performed when he was diagnosed with a possible acute Q fever. A pre‐existing undetected mitral valve regurgitation may have gone undiagnosed and predisposed the patient to eventually develop endocarditis.

The histopathological findings of non‐necrotizing gastric granulomas were striking. Non‐necrotizing granulomas may be seen in a variety of medical conditions including sarcoidosis, Crohn's disease or granulomatous mycobacterial or fungal infections [5].

Histological studies of Q fever based on infected organs biopsies (aortic wall, liver, and bone marrow) have demonstrated a distinctive type of granuloma, typically appearing as a “doughnut” granuloma, which has a central lipid vacuole surrounded by a dense fibrin ring [6, 7, 8]. We did not find any publications reporting Q fever‐related gastric granulomas, typical fibrin ring‐type granulomas, or non‐necrotizing granulomas. The discovery of granulomas in this atypical location for Q fever manifestations led us to initially consider other diagnoses.

Finally, *C. burnetii* infection is widely present in Spain [9, 10] and in the Balearic islands where a large series of Q fever has been reported [11], showing that it is a common entity that could be underdiagnosed.

## 6 Conclusion

Non‐specific symptoms such as weight loss and anorexia may be related to early stages of chronic Q fever and granulomas in atypical locations such as the stomach may be present. A high index of clinical suspicion is required to diagnose chronic Q fever when endocarditis appears as a late symptom.

## Author Contributions

**Ana Elena Lago‐Rodriguez:** conceptualization, data curation, investigation, methodology, project administration, writing – original draft, writing – review and editing. **Pablo Arturo Fraile‐Ribot:** data curation, investigation, methodology, supervision, writing – review and editing. **Isabel Amengual‐Antich:** data curation, formal analysis, investigation, supervision, writing – review and editing. **Francisco Gual‐Capllonch:** data curation, investigation, validation, writing – review and editing. **Mercedes Garcia‐Gasalla:** conceptualization, data curation, formal analysis, methodology, project administration, writing – original draft, writing – review and editing.

## Consent

A signed patient informed consent to publish the details of the clinical case has been obtained.

## Conflicts of Interest

The authors declare no conflicts of interest.

## Data Availability

Data sharing is not applicable to this article as no new data were created or analyzed in this study.
